# Effects of intravitreal injection of ranibizumab and aflibercept for branch retinal vein occlusion on the choroid: a retrospective study

**DOI:** 10.1186/s12886-022-02685-4

**Published:** 2022-11-30

**Authors:** Shuta Kishishita, Yoshihito Sakanishi, Shu Morita, Moe Matsuzawa, Ayumi Usui-Ouchi, Nobuyuki Ebihara

**Affiliations:** Department of Ophthalmology, Juntendo University Urayasu Hospital, 2-1-1 Tomioka, 279-0021 Urayasu, Chiba Japan

**Keywords:** Aflibercept, Branch retinal vein occlusion, Choroid, Intravitreal injection, Ranibizumab

## Abstract

**Background:**

Macular edema is found in more than half of branch retinal vein occlusion (BRVO) cases, leading to visual loss in most of these cases. Intravitreal injection of anti-vascular endothelial growth factor is currently the standard treatment for macular edema due to BRVO (BRVO-ME). The difference in the effects of aflibercept and ranibizumab on the choroid in BRVO-ME is unknown. Therefore, we analyzed the effects of intravitreal injection of ranibizumab and aflibercept on BRVO-ME.

**Methods:**

We retrospectively observed changes in choroidal thickness in the subfoveal region in 36 patients with BRVO-ME who visited the Department of Ophthalmology at the Juntendo University Urayasu Hospital. The patients were treated with intravitreal injection of aflibercept or ranibizumab and followed up for 12 months or more.

**Results:**

The observed point bifurcated into the affected and non-affected sides 500 μm from the fovea. The central macular thickness (CMT) and subfoveal choroidal thickness (SFCT) were 564.2 ± 268.5 μm and 228.8 ± 50.1 μm, respectively, in the ranibizumab group (16 patients, 16 eyes) and 542.4 ± 172.5 μm and 246.1 ± 59.1 μm, respectively, in the aflibercept group (20 patients, 20 eyes). The changes in CMT at 12 months were 324.0 ± 262.6 μm and 326.55 ± 187.2 μm in the ranibizumab and aflibercept groups, respectively, with no significant difference (*p *= 0.97). Similarly, the changes in SFCT over 12 months were not significant between the groups (ranibizumab, 41.9 ± 33.0 μm; aflibercept, 43.8 ± 43.8 μm, *p* = 0.89).

**Conclusion:**

The effects of ranibizumab and aflibercept on choroidal thickness in BRVO-ME were the same regardless of the site. Although BRVO is a retinal disease, we hope that we can further explore the mechanism of BRVO-ME by observing changes in the choroid in the future.

## Background

Branch retinal vein occlusion (BRVO) is a condition in which retinal hemorrhage occurs due to obstruction of the retinal vein. BRVO often occurs at the arteriovenous crossroads. Since the retinal arteries and veins have an adventitia at the intersection, arteriosclerotic changes compress the retinal veins and cause stenosis of the lumen. As a result, it is considered that a thrombus is formed due to impaired blood flow and causes venous obstruction. Venous occlusion increases venous return pressure, and macular edema occurs due to leakage into the retina from the foveal capillary and vascular permeability. Macular edema is found in more than half of BRVO cases and is the most important cause of visual loss due to BRVO [1]. Intravitreal injection of anti-vascular endothelial growth factor (VEGF) is currently the standard treatment for macular edema due to BRVO (BRVO-ME). A BRVO study reported an increase in visual acuity in patients with corrected visual acuity of 0.5 or less after monthly administration of ranibizumab for 6 months and a decrease in mean central macular thickness (CMT), and administration as needed (PRN) improved visual acuity even after 6 to 12 months [2]. The HORIZON trial reported a sustained improvement in visual acuity even 24 months after the administration of ranibizumab [3]. In addition, Sakanishi et al. [4] administered an intravitreal injection of ranibizumab (IVR) for BRVO and central retinal vein occlusion (CRVO) with macular edema and reported an improvement of visual acuity and foveal retinal thickness after drug administration. These studies showed that intravitreal injection of anti-VEGF drugs led to long-term improvements in visual acuity, and the dose efficacy increased with time [1–4]. Intravitreal injection of anti-VEGF drugs has become the standard treatment for retinal diseases such as age-related macular degeneration (AMD) and BRVO-ME [5, 6]. However, effects on the choroid have also been reported [7–10]. Tsuiki et al. reported that a single dose of ranibizumab in 36 eyes with BRVO-ME and CRVO significantly reduced the mean foveal vein thickness [7]. Additionally, a reduction in choroidal thickness (CT) following IVR or intravitreal injection of aflibercept (IVA) for AMD has been reported [8–10]. In AMD, the degree of influence on CT depends on the type of anti-VEGF drug. Koizumi et al. [9] reported that when aflibercept and ranibizumab were administered to AMD patients 3 times in 3 months, the rate of reduction in mean subfoveal CT (SFCT) was significantly greater in the aflibercept group. In addition, CT was significantly decreased in eyes with reticular pseudodrusen (RPD) compared to healthy eyes and those with drusen. Therefore, RPD seems to be a consequence of an alteration in the choroidal vascularity, resulting in severe ischemia and excessive hypoxia and inducing a greater risk of late AMD than that seen in healthy eyes and those with drusen [10]. However, the difference in the effects of aflibercept and ranibizumab on the choroid in BRVO-ME is unknown. Therefore, we analyzed the effects of IVR and IVA on the choroid in BRVO-ME patients.

## Methods

**Fig. 1:**
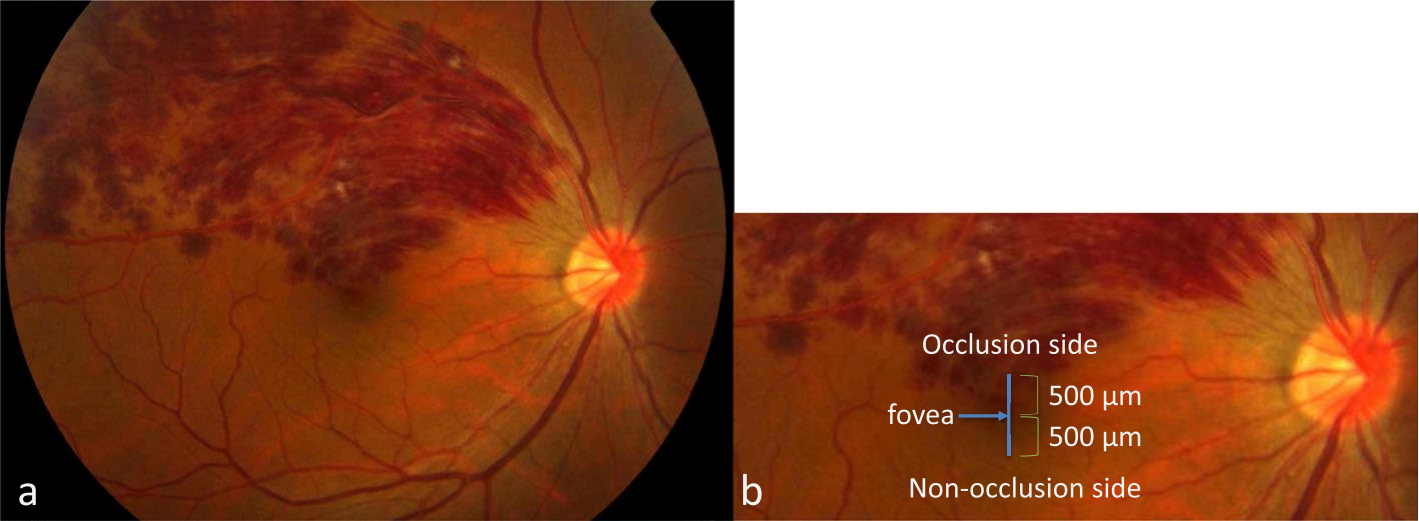
The measurement position of this study. Central macular thickness (CMT), subfoveal choroidal thickness (SFCT), and choroidal thickness (CT) on the affected and non-affected sides were measured at a distance of 500 μm in the longitudinal direction from the fovea. **A** Overall view of the major branch retinal vein occlusion. **B** Magnified image of the macula

Among the untreated BRVO-ME cases who visited the Department of Ophthalmology at Juntendo University Urayasu Hospital in the last 5 years, we selected those who were treated with IVA or IVR in the acute phase within 6 months after disease onset and could be followed up for 12 months or more. IVA cases were chosen in the first half of the observation period, and IVR cases were chosen in the second half of the period. Additionally, we excluded cases in which the injected drugs were switched or retinal photocoagulation (PC) and grid PC were performed on the avascular field. We performed a medical examination a week after the first dose of IVR or IVA. The first administration was performed, and re-administration was performed PRN at monthly consultations. The initial doses of IVA and IVR were 2.0 mg and 0.5 mg, respectively, with a similar PRN dose. The re-administration standard was prolonged CMT or recurrence beyond 300 μm. CT changes were not included in the criteria. Optical coherence tomography (Cirrus HD; Carl Zeiss Meditec, Jena, Germany) was used to measure retinal thickness and CT; the 5-line HD mode was used to measure CMT, and the 5-line HD enhanced depth imaging mode was used to measure CT. We used one of the 5 lines, which is most suitable for measuring CMT or SFCT. The reticulum thickness was defined as the distance from the surface of the inner limiting membrane to the inner line of the retinal pigment epithelium, and CT was defined as the distance from the outer line of the retinal pigment epithelium to the transition to the sclera [11]. Furthermore, visual acuity was measured by a decimal visual acuity meter with a 5-m Landolt ring, which was converted into logMAR. CMT, SFCT, and CT on the affected and non-affected sides were measured at 500 μm in the longitudinal direction from the fovea (Fig. 1). All cases were measured between 14:00 and 16:00 to rule out the effects of diurnal variation. Based on the medical records, the amount of change in retinal thickness and CT between the two drug-treated groups were retrospectively compared and examined. Additionally, the correlation between the amount of change in retinal thickness and CT was examined for each drug.

This study was approved by the Ethics Committee of Juntendo University of Urayasu Hospital and conducted in accordance with the Declaration of Helsinki in 2013. We also obtained informed consent. Statistical studies were conducted using SPSS 26 for Mac statistical software (IBM, Armonk, NY). The Mann–Whitney U test was used to compare continuous variables, and Fisher’s exact test was used to compares multi-variable entities. Pearson’s product-moment correlation was used to analyze the correlation between retinal thickness and CT. Statistical significance was established at *p* < 0.05.

## Results

We analyzed 16 eyes from 16 patients in the IVR group and 20 eyes from 20 patients in the IVA group. The case background of each group is shown in Table 1. The average age of patients with IVR was 67.8 ± 9.9 years, and that of those with IVA was 70.0 ± 7.1 years. There were 8 males and 8 females with IVR, and 10 males and 10 females in IVA cases. The average pre-injection vision acuity (logMAR) of IVR cases was 0.53 ± 0.34, and that of IVA cases was 0.40 ± 0.22. The average pre-injection CMT of IVR cases was 564.2 ± 268.5 μm, and that of IVA cases was 542.4 ± 172.5 μm. The average pre-injection SFCT of IVR cases was 228.8 ± 50.1 μm, and that of IVA cases was 246.1 ± 59.1 μm. There were no significant differences between the two groups among all variables. The average number of injections in the IVR and IVA groups were 2.5 ± 1.7 and 2.1 ± 1.1, respectively.

**Table 1:** Characteristics of the ranibizumab and aflibercept groups

	IVR (16 cases, 16 eyes)	IVA (20 cases, 20 eyes)	P-value
Age (years)	67.8 ± 9.9	70.0 ± 7.1	0.50
Sex (male/female)	8/8	10/10	0.79
Pre-injection visual acuity (logMAR)	0.53 ± 0.34	0.40 ± 0.22	0.64
Duration from onset (months)	2.0 ± 1.4	2.0 ± 1.6	0.50
Pre-injection CMT (µm)	564.2 ± 268.5	542.4 ± 172.5	0.97
Pre-injection SFCT (µm)	228.8 ± 50.1	246.1 ± 59.1	0.27
Major/Macula	10/6	16/4	0.32
Hypertension presence/absence	10/6	7/13	0.31

*IVR* intravitreal injection of ranibizumab, *IVA* intravitreal injection of aflibercept, *logMAR* logarithm of the minimum angle, *CMT* central macular thickness, *SFCT* subfoveal choroidal thickness.

**Fig. 2:**
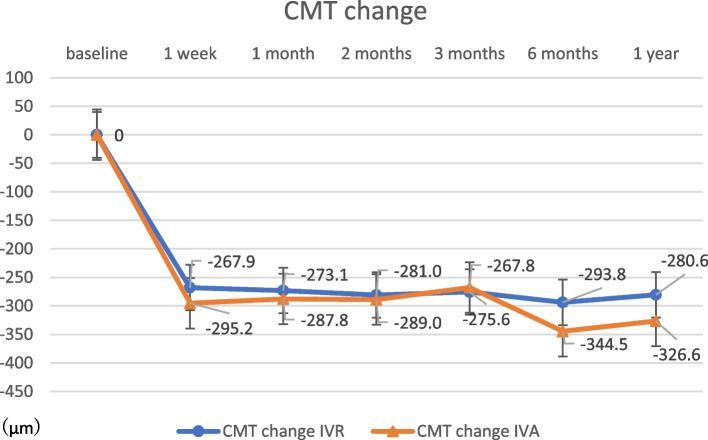
Change in CMT in the IVR and IVA groups. There was no significant change in CMT at 12 months post-treatment in the IVR group or in the IVA group (*p* = 0.53). CMT, central macular thickness; IVR, intravitreal injection of ranibizumab; IVA, intravitreal injection of aflibercept

**Fig. 3:**
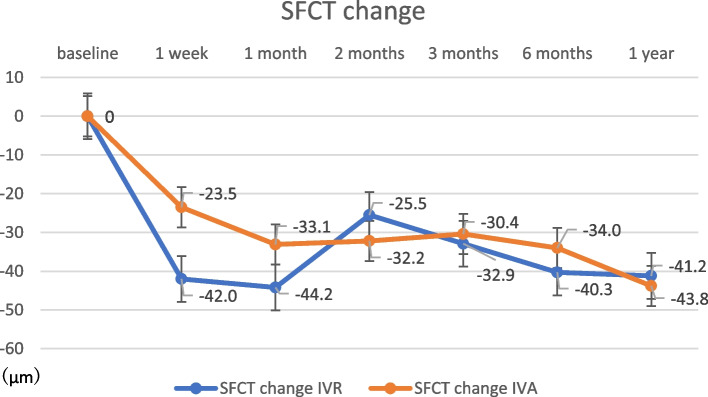
Change in SFCT in the IVR and IVA groups. There was no significant change in SFCT at 12 months post-treatment in the IVR group or in the IVA group (*p* = 0.37). SFCT, subfoveal choroidal thickness; IVR, intravitreal injection of ranibizumab; IVA, intravitreal injection of aflibercept

**Fig. 4:**
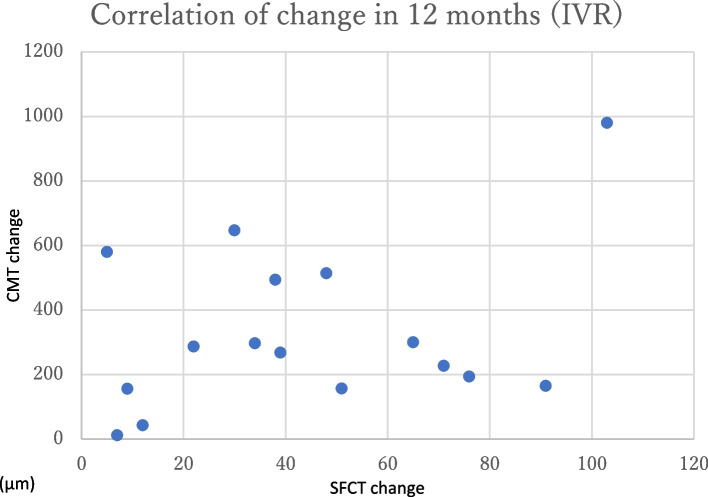
Relationship between the amount of change in CMT and SFCT in the IVR group. No correlation was found between the amount of change in CMT and SFCT in the IVR group (correlation coefficient = 0.24, *p* = 0.23, respectively). CMT, central macular thickness; SFCT, subfoveal choroidal thickness; IVR, intravitreal injection of ranibizumab

**Fig. 5:**
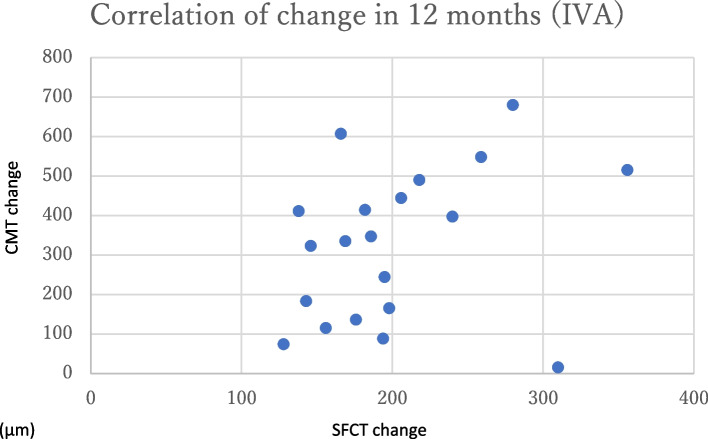
Relationship between the amount of change in CMT and SFCT in the IVA group. No correlation was found between the amount of change in CMT and SFCT in the IVA group (correlation coefficient = 0.22, *p* = 0.21, respectively). CMT, central macular thickness; SFCT, subfoveal choroidal thickness; IVA, intravitreal injection of aflibercept

**Fig. 6:**
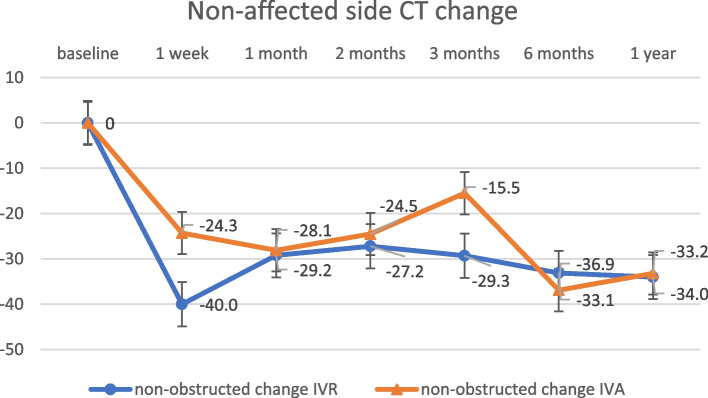
The level of change in CT on the non-affected side in the IVR and IVA groups. There were no significant differences between the two groups (*p* = 0.73). CT, choroidal thickness; IVR, intravitreal injection of ranibizumab; IVA, intravitreal injection of aflibercept

**Fig. 7:**
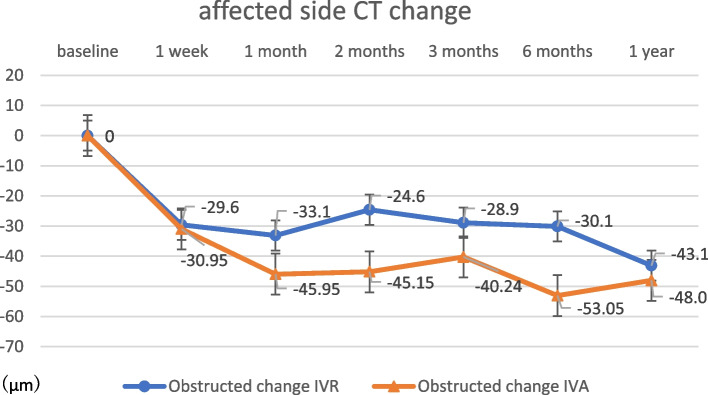
The level of change in CT on the affected side in the IVR and IVA groups. There were no significant differences between the two groups (*p* = 0.95). CT, choroidal thickness; IVR, intravitreal injection of ranibizumab; IVA, intravitreal injection of aflibercept

The change in CMT at 12 months post-treatment was 324.0 ± 262.6 μm in the IVR group and 326.6 ± 187.2 μm in the IVA group (Fig. 2), with no significant difference (*p* = 0.97). The change in SFCT at 12 months post-treatment was 41.9 ± 33.0 μm in the IVR group and 43.8 ± 43.8 μm in the IVA group (Fig. 3), with no significant difference (*p* = 0.89). There were no significant differences in any variable at any post-treatment duration. No correlation was found between the amount of change in CMT and SFCT in either group at 1, 2, 3, 6, or 12 months (Figs. 4 and 5; CMT, correlation coefficient = 0.24, *p* = 0.23; SFCT, correlation coefficient = 0.22, *p *= 0.21, respectively). The level of change in CT on the affected side was 43.1 ± 35.0 μm in the IVR group and 48.0 ± 41.6 μm in the IVA group, and that on the non-affected side was 34.0 ± 36.2 μm in the IVR group and 33.2 ± 45.1 μm in the IVA group (Figs. 6 and 7). There were no significant differences between the two groups in either side (*p* = 0.73 and *p* = 0.95, respectively). As a result, there was no difference in the change in CT between the two drug-treated groups at any of the sites.

## Discussion

In this study, we found no significant differences in the change in CT between IVR- and IVA-treated BRVO-ME patients. It has been reported that the SFCT in eyes with BRVO becomes significantly thicker than in healthy eyes [13], which is thought to be caused by increased choroidal vascular permeability due to increased intraocular VEGF concentration [7]. Furthermore, a higher VEGF concentration has been reported to promote nitric oxide production, increasing CT [14]. It is also known that CT changes after anti-VEGF treatment. A single administration of ranibizumab to BRVO-ME patients and macular edema patients with CRVO has been known to significantly reduce average SFCT [7]. In the present study, the CT in BRVO-ME showed a similar decrease. With respect to the comparison between two anti-VEGF treatments for BRVO-ME, a prospective study comparing the effects of ranibizumab and aflibercept on CRVO reported no significant difference in visual acuity or change in CMT between the two groups [15]. Additionally, no differences were found in visual acuity or CMT with either drug in non-ischemic BRVO [16]. These reports compared the CMT in BRVO between ranibizumab and aflibercept but did not describe the difference in CT. However, in AMD, Koizumi et al. [9] reported a significant reduction in mean SFCT in the aflibercept-treated group when aflibercept and ranibizumab were continuously administered for 3 months each. Similarly, Kaya [17] reported that IVR and IVA treatment for AMD reduced CT, and the reduction rate was significantly greater in IVA-treated patients. In other words, while IVR and IVA differ in their rates of decrease in SFCT in AMD, they do not differ in BRVO, as our results show. This is because the main pathological condition in AMD is in the choroid, while it is in the retina in BRVO. Compared to ranibizumab, aflibercept is known to have a higher degree of invasion in the choroid, and this difference causes variable effects on the choroid in AMD. However, in BRVO, the choroidal changes are secondary, caused by the increased expression of VEGF from the retina, and there is no difference in the depth of invasion of the retina, which is the main pathological condition. As a result, we found no significant difference in CT in our study. Additionally, as reported by Koizumi et al. [9] and Kaya [17], the drugs were injected three times during the introductory period. However, in our study, only one injection was administered during the introductory period. It is possible that differences between drug efficacies become apparent with multiple consecutive injections. However, in clinical practice in Japan, a single drug administration is performed during the introductory period. A multiple-administration introduction period is not suitable for actual clinical practice; therefore, in this study, we chose the single-dose regimen during the introductory period [18, 19].

This study was limited in that it was a single-institution retrospective study with a small number of patients. Therefore, to confirm our results, a retrospective study involving multiple institutions and a large number of patients is warranted. In addition, we previously reported choroidal thinning after thickening (re-thinning) during recurrence and re-injection after first IVA for BRVO in the fovea centralis in both the affected and non-affected sides [20]. It has been shown that there is a close relationship between changes in CT and the timing of recurrence of macular edema. Although BRVO is a retinal disease, we can look at it from a new perspective by observing the changes in the choroid.

## Conclusion

In conclusion, we found that the effects of IVR and IVA treatment on CT in BRVO-ME patients were the same at all sites. Although BRVO is a retinal disease, we can identify and observe the choroidal changes, thereby hoping to explore the mechanism of BRVO-ME. We hope that this different point of view can prevent many complications in BRVO patients and improve their quality of life in the future.

## Data Availability

The datasets generated and analyzed during the current study are available from corresponding author on reasonable request.

## Abbreviations

AMD: Age-related macular degeneration
BRVO: Branch retinal vein occlusion
CMT: Central macular thickness
CT: Choroidal thickness
IVA: Intravitreal injection of aflibercept
IVR: Intravitreal injection of ranibizumab
ME: Macular edema
SFCT: Subfoveal choroidal thickness
VEGF: Vascular endothelial growth factor

## References

[1] Ehlers JP, Kim SJ, Yeh S, Thorne JE, Mruthyunjaya P, Schoenberger SD (2017). Therapies for macular edema associated with branch retinal vein occlusion: a report by the American Academy of Ophthalmology. Ophthalmology.

[2] Campochiaro PA, Heier JS, Feiner L, Gray S, Saroj N, Rundle AC (2010). Ranibizumab for macular edema following branch retinal vein occlusion: six-month primary end point results of a phase III study. Ophthalmology.

[3] Heier JS, Campochiaro PA, Yau L, Li Z, Saroj N, Rubio RG (2012). Ranibizumab for macular edema due to retinal vein occlusions: long-term follow-up in the HORIZON trial. Ophthalmology.

[4] Sakanishi Y, Ouchi A, Ito R, Ebihara N (2016). [Six months outcome in patients with macular edema due to retinal vein occlusion treated with ranibizumab]. Nippon Ganka Gakkai zasshi.

[5] Rosenfeld PJ, Brown DM, Heier JS, Boyer DS, Kaiser PK, Chung CY (2006). Ranibizumab for neovascular age-related macular degeneration. N Engl J Med.

[6] Lalwani GA, Rosenfeld PJ, Fung AE, Dubovy SR, Michels S, Feuer W (2009). A variable-dosing regimen with intravitreal ranibizumab for neovascular age-related macular degeneration: year 2 of the PrONTO Study. Am J Ophthalmol.

[7] Tsuiki E, Suzuma K, Ueki R, Maekawa Y, Kitaoka T (2013). Enhanced depth imaging optical coherence tomography of the choroid in central retinal vein occlusion. Am J Ophthalmol.

[8] Yamazaki T, Koizumi H, Yamagishi T, Kinoshita S (2012). Subfoveal choroidal thickness after ranibizumab therapy for neovascular age-related macular degeneration: 12-month results. Ophthalmology.

[9] Kizumi H, Kano M, Yamamoto A, Saito M, Maruko I, Sekiryu T (2016). Subfoveal choroidal thickness during aflibercept therapy for neovascular age-related macular degeneration: twelve-month results. Ophthalmology.

[10] Viggiano P, Toto L, Ferro G, Evangelista F, Porreca A, Mastropasqua R (2022). Choroidal structural changes in different intermediate AMD patterns. Eur J Ophthalmol.

[11] Hasegawa T, Kawano T, Maruko I, Koizumi H, Iida T (2018). Clinical findings of eyes with macular edema associated with branch retinal vein occlusion refractory to ranibizumab. Retina.

[12] Kang HM, Kwon HJ, Yi JH, Lee CS, Lee SC (2014). Subfoveal choroidal thickness as a potential predictor of visual outcome and treatment response after intravitreal ranibizumab injections for typical exudative age-related macular degeneration. Am J Ophthalmol.

[13] Kim KH, Lee DH, Lee JJ, Park SW, Byon IS, Lee JE (2015). Regional choroidal thickness changes in branch retinal vein occlusion with macular edema. Ophthalmologica.

[14] Mrejen S, Spaide RF (2013). Optical coherence tomography: imaging of the choroid and beyond. Surv Ophthalmol.

[15] Sashin Y, Ito Y, Fujikawa M, Sawada T, Ohji M (2017). Comparison between ranibizumab and aflibercept for macular edema associated with central retinal vein occlusion. Jpn J Ophthalmol.

[16] Pichi F, Elbarky AM, Elhamaky TR (2019). Outcome of “treat and monitor” regimen of aflibercept and ranibizumab in macular edema secondary to non-ischemic branch retinal vein occlusion. Int Ophthalmol.

[17] Kaya F (2017). Change in choroidal thickness after intravitreal injection for treatment of neovascular age-related macular degeneration: ranibizumab versus aflibercept. J Fr Ophtalmol.

[18] Sato M, Minami S, Nagai N, Suzuki M, Kurihara T, Shinojima A (2020). Association between axial length and choroidal thickness in early age-related macular degeneration. PLoS ONE.

[19] Sakanishi Y, Lee A, Usui-Ouchi A, Ito R, Ebihara N (2016). Twelve-month outcomes in patients with retinal vein occlusion treated with low-frequency intravitreal ranibizumab. Clin Ophthalmol.

[20] Sakanishi Y, Tamaki K, Mashimo K, Sakuma T, Ebihara N (2021). Relationship between recurrence of macular edema due to branch retinal vein occlusion and changes in choroidal thickness. Ophthalmic Res.

